# Population-Based Influenza Vaccine Effectiveness Against Laboratory-Confirmed Influenza Infection in Southern China, 2023–2024 Season

**DOI:** 10.1093/ofid/ofae456

**Published:** 2024-08-21

**Authors:** Xīn Gào, Yexiang Sun, Peng Shen, Jinxin Guo, Yunpeng Chen, Yueqi Yin, Zhike Liu, Siyan Zhan

**Affiliations:** Department of Epidemiology and Biostatistics, School of Public Health, Peking University, Beijing, China; Key Laboratory of Epidemiology of Major Diseases, Peking University, Ministry of Education, Beijing, China; Yinzhou District Center for Disease Control and Prevention, Ningbo, China; Yinzhou District Center for Disease Control and Prevention, Ningbo, China; Department of Epidemiology and Biostatistics, School of Public Health, Peking University, Beijing, China; Key Laboratory of Epidemiology of Major Diseases, Peking University, Ministry of Education, Beijing, China; Yinzhou District Center for Disease Control and Prevention, Ningbo, China; Yinzhou District Center for Disease Control and Prevention, Ningbo, China; Department of Epidemiology and Biostatistics, School of Public Health, Peking University, Beijing, China; Key Laboratory of Epidemiology of Major Diseases, Peking University, Ministry of Education, Beijing, China; Department of Epidemiology and Biostatistics, School of Public Health, Peking University, Beijing, China; Key Laboratory of Epidemiology of Major Diseases, Peking University, Ministry of Education, Beijing, China; Center for Intelligent Public Health, Institute for Artificial Intelligence, Peking University, Beijing, China; Research Center of Clinical Epidemiology, Peking University Third Hospital, Beijing, China

**Keywords:** influenza, post-COVID-19, real-world data, test-negative design, vaccine effectiveness

## Abstract

**Background:**

In China, the 2022–2023 influenza season began earlier and was characterized by higher levels of influenza activity and co-circulation of various respiratory pathogens compared with seasons before the coronavirus disease 2019 (COVID-19) pandemic. Timely and precise estimates of influenza vaccine effectiveness (IVE) against infections can be used to guide public health measures.

**Methods:**

A test-negative study was conducted to estimate IVE against laboratory-confirmed influenza using data from the CHinese Electronic health Records Research in Yinzhou (CHERRY) study that prospectively integrated laboratory, vaccination, and health administrative data in Yinzhou, southern China. We included patients who presented influenza-like illness and received nucleic acid tests and/or antigen tests between October 2023 and March 2024. Estimates of IVE were adjusted for age, gender, month of specimen submitted, chronic comorbidities, and hospitalization status.

**Results:**

A total of 205 028 participants, including 96 298 influenza cases (7.6% vaccinated) and 108 730 influenza-negative controls (13.4% vaccinated), were eligible for this analysis. The estimates of IVE were 49.4% (95% CI, 47.8%–50.9%), 41.9% (95% CI, 39.8%–44.0%), and 59.9% (95% CI, 57.9%–61.9%) against overall influenza, influenza A, and influenza B, respectively. A lower IVE was observed for individuals aged 7–17 years (38.6%), vs 45.8% for 6 months–6 years, 46.7% for 18–64 years, and 46.1% for ≥65 years. Vaccination reduced the risk of infection by 44.4% among patients with chronic comorbidities. IVEs varied by epidemic weeks with the changes in influenza activity levels and the switch of dominant influenza strains.

**Conclusions:**

Influenza vaccination in the 2023–2024 season was protective against infection for the entire population.

Seasonal influenza is an acute respiratory infection caused by influenza A and B viruses, which leads to a significant disease burden as well as huge economic costs [1]. In China, the seasonal circulation patterns of influenza have changed over the past 4 years, resulting from the implementation and relaxation of rigorous nonpharmaceutical interventions (NPIs) against COVID-19 [2]. In late 2023, acute respiratory infections substantially upsurged across China due to a high level of co-circulation of influenza with various known pathogens including *Mycoplasma* pneumonia, respiratory syncytial virus, and adenoviruses [3–5], which has drawn great attention from the World Health Organization (WHO) [6]. Typically, influenza activity in southern China has 2 peaks throughout the year, a distinct peak in the summer (from June to August) and a less marked peak in the winter (from January to February) [7]. However, an earlier onset of influenza activity was observed during the 2023–2024 winter season compared with previous years [8, 9]. Moreover, levels of influenza activity were higher in southern provinces, where the population accounts for more than half of the total population of China, than in northern provinces [8].

Influenza vaccination is a cornerstone of public health measures, significantly reducing the burden of influenza-related morbidity and mortality, especially in children under age 5 and elderly adults [10–12]. In addition, research has consistently shown that influenza vaccines are effective in reducing the risk of major adverse cardiovascular events and mortality [13, 14]. The influenza vaccines used in China during the 2023–2024 epidemic season included the trivalent inactivated influenza vaccine (IIV3), the quadrivalent inactivated influenza vaccine (IIV4), and the trivalent live attenuated influenza vaccine (LAIV3). However, due to the constant antigenic shift and drift of influenza viruses, particularly influenza A virus, vaccines need to be reformulated before each season. Influenza vaccine effectiveness (IVE) may not be optimal in cases of mismatch between vaccine strains and circulating viruses [1]. Therefore, it is important to track annual estimates of IVE to ensure that vaccines remain effective against evolving influenza strains and to further inform influenza prevention policies and strategies, for instance, making recommendations for future vaccine compositions by health care authorities [15].

Given the differences between the 2023–2024 influenza season and previous seasons and the unpredictability of influenza antigenic variations, timely and precise evaluation of the effectiveness of the influenza vaccine is of particular importance. However, to our knowledge, only a few small-sample studies have investigated IVE after lifting COVID-19 restrictions in China [16, 17]. Overall estimates of IVE in the 2023–2024 season and dynamic IVE rates along the course of the epidemic have not been reported in the Chinese population. In Yinzhou, southern China, laboratory, vaccination, and health administrative data are available for the entire registered population and have been used to establish a longitudinal population-based ambidirectional cohort [18], in which the effectiveness and safety of various vaccines were evaluated [19, 20]. This study aims to provide granular and timely estimates of overall and weekly IVE in the 2023–2024 season in southern China using comprehensive health care data from the cohort.

## METHODS

### Data Sources

Influenza sentinel surveillance data from southern China were obtained from weekly reports from the Chinese National Influenza Center (CNIC) [8]. Geographically, southern China includes the region to the south of the Qinling Mountains-Huaihe River Line, which covers 15 provinces including Shanghai, Jiangsu, Zhejiang, Anhui, Fujian, Jiangxi, Hubei, Hunan, Guangdong, Guangxi, Hainan, Chongqing, Sichuan, Guizhou, and Yunnan. Each surveillance week, the CNIC releases the numbers of influenza-like illness (ILI) specimens and numbers of influenza strains isolated from respiratory specimens of ILI cases, which are determined by nucleic acid amplification test (NAAT).

IVE estimations were based on data from the CHinese Electronic health Records Research in Yinzhou (CHERRY) study, which is an ongoing longitudinal ambidirectional cohort. This cohort was preliminary designed to investigate risk factors for cardiovascular disease, and the detailed cohort information was reported in a published protocol [18]. The CHERRY cohort has also been used to investigate the real-world safety and effectiveness of vaccines [21–23]. Briefly, the CHERRY cohort was established based on a real-time updated health care data platform covering 98% of the 1.2 million registered residents of Yinzhou, which is an economically developed region located in southeast China (comparable to New Orleans in the USA). This platform integrated multisource administrative databases including general demographic characteristics, check-up information, inpatient, outpatient, and emergency electronic medical records, pathogen laboratory testing data, immunization registry, and death registry. The CHERRY cohort included all registered individuals since January 1, 2009, and updated vital status, clinical outcomes, and claims data for all cohort participants annually until death or moving to another province. This study was conducted following the tenets of the Declaration of Helsinki, Good Pharmacoepidemiology Practice Guidelines, and local laws. This study was approved by the institutional review board and human research ethics committee of the Yinzhou District Center for Disease Control and Prevention. Informed consent was waived as all data were de-identified, anonymized, and accessible only in an isolated local network.

### IVE Study Design and Study Population

A test-negative case–control design was used to estimate the IVE for the 2023–2024 influenza season. Positivity crossed the 10% threshold in week 38/2023, indicating the start of the seasonal epidemic (Supplementary Figure 1). In this study, patients who presented with ILI and received testing for influenza between week 38 of 2023 and week 12 of 2024 were included in inpatient, outpatient, and emergency settings. ILI is considered a fever (axillary temperature ≥38℃) accompanied by a cough or sore throat.

Influenza vaccination exposure for IVE estimation was defined as receipt of at least 1 dose of the vaccine in the CHERRY cohort within the vaccination campaign period (August 1, 2023, to March 11, 2024). Influenza vaccination status of the current influenza season was available for all included participants. Subjects who received an influenza vaccine <14 days before the specimen collection date were excluded.

According to the Chinese guidelines for influenza, both NAATs and rapid antigen tests (RATs; including Colloidal Gold and immunofluorescence assay) can be used for influenza diagnosis during influenza season [24, 25]. Cases were defined based on the positive influenza testing results during the season, and controls were individuals who tested negative throughout the season. The first NAAT and/or RAT results and dates were used in the current analyses.

### Covariate Ascertainment

Birth date, gender, and calendar month of specimen collection were available in the cohort. Age was determined at the time the specimen was submitted. Participants were regarded as being in the inpatient setting if specimen collection dates were between the admit date and discharge date. Based on the 10th Revision of the International Statistical Classification of Diseases (ICD-10) records, participants diagnosed with at least 1 of the following diseases were considered to have a chronic comorbidity: cancer, hypertension, cerebrovascular diseases, cardiovascular diseases, diabetes, congestive heart failure, chronic obstructive pulmonary disease (COPD), chronic kidney disease, and neural degenerative diseases (Supplementary Table 1).

### Statistical Analyses

The influenza positivity rate was calculated by dividing the number of patients with positive test results by the total number of patients who had NAATs or RATs for influenza tests. Baseline characteristics of the study population were categorized and were described as numbers and percentages. Cohen's *h* was calculated to measure the differences in proportions. The differences are negligible, small, medium, and large if *h* values were <0.20, 0.20–0.49, 0.50–0.79, and ≥0.80, respectively. The chi-square test was employed to test for statistical significance by comparing proportions.

Odds ratios were derived from univariate and multivariate logistic regression for the IVE calculation as (1 ‒ odds ratio) × 100%. The multivariate model was adjusted for age, gender, calendar month of specimen collection, hospitalization status (inpatient or non-inpatient), and presence of chronic comorbidity. The overall IVE and IVEs for influenza A and B were estimated. Subgroup IVEs were evaluated in different age groups (6 months–6 years, 7–17 years, 18–64 years, and ≥65 years), patients with chronic comorbidities, and different hospitalization statuses (inpatient and outpatient/emergency). We further applied Firth's penalized logistic regression to minimize small-sample bias in the analyses estimating weekly IVE [26].

Several sensitivity analyses were performed. We estimated the effectiveness of midseason vaccination, which was defined as a vaccination date later than the start of the influenza season. We further restricted the analyses to the periods when positivity rates of influenza A and influenza B were >10%. IVEs were also estimated according to the influenza testing types, namely, NAAT and RAT.

All statistical hypothesis tests were 2-tailed, and *P* values <.05 were considered statistically significant. SAS software (version 9.4; SAS Institute Inc., Cary, NC, USA) was used to perform Cohen's *h* calculation, the chi-square test, and logistic regression models.

## RESULTS

### Features of Influenza Activity in Southern China

**Figure 1.**
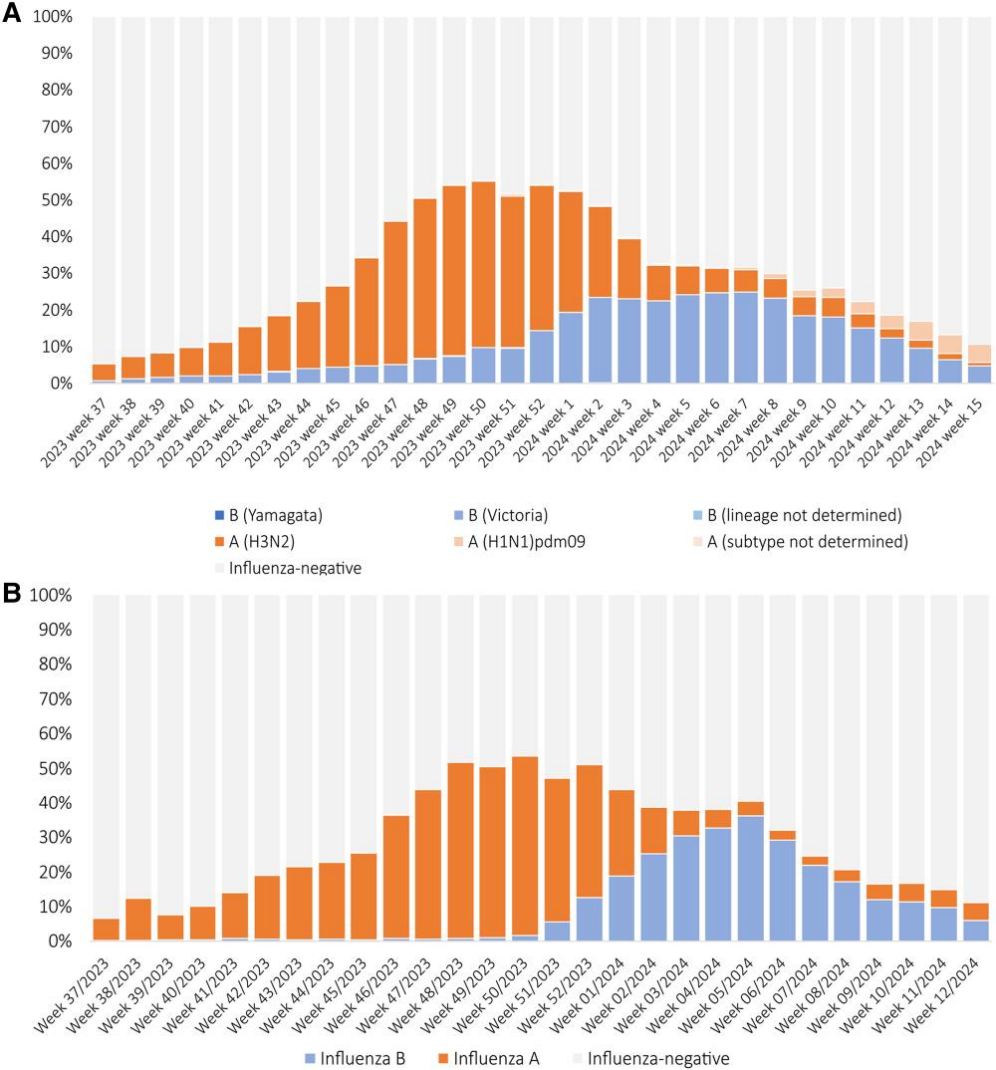
Weekly positivity rate from week 37/2023 to week 12/2024, 2023–2024 influenza season, in sentinel hospitals in southern provinces (A) and in Yinzhou, Zhejiang province, southern China (B).

Figure 1A presents weekly percentages of influenza strains in submitted specimens from sentinel hospitals in 15 southern provinces between September 4, 2023, and April 13, 2024. The overall percentage of influenza-positive specimens peaked at 55.2% in week 50/2023. Influenza A (H3N2) was the dominant strain from the beginning of the influenza season until week 3 of 2024, in which the B/Victoria lineage became the dominant strain. The highest positivity rates were observed in week 49/2023 and week 7/2024 for A (H3N2; 45.6%) and B/Victoria (24.9%), respectively. The percentage of A(H1N1)pdm09-positive specimens started increasing in late March. In week 15/2024, A(H1N1)pdm09 was the most detected strain and showed an increasing trend.

In Yinzhou, the weekly influenza positivity rates were calculated in tested ILI cases (inpatient and outpatient/emergency) from the CHERRY cohort between September 4, 2023, and March 25, 2024 (Figure 1B; Supplementary Figure 1). The peak influenza positivity rate was 53.4% in week 50/2023. During the first 15 weeks of the season, influenza A strains were the most frequently detected viruses and peaked in week 50/2023 with a positivity rate of 51.8%. The positivity rate for influenza B gradually increased and reached a peak of 36.3% in week 5/2024.

### Characteristics of the IVE Study Population in Yinzhou

Overall, 205 028 participants were included between September 4, 2023, and March 25, 2024, including 108 730 test-negative controls and 96 298 test-positive cases. The characteristics of controls and cases are shown in Table 1. Among influenza test–negative controls, 9.0% were elderly, vs 2.8%, 3.2%, and 1.7% of overall influenza, virus A, and virus B cases, respectively. More females were included in controls (54.5%) compared with cases of overall influenza (50.6%), virus A (50.7%), and virus B (50.1%). The percentages of vaccinated individuals in controls, overall influenza cases, influenza A cases, and influenza B cases were 13.4%, 7.6%, 8.4%, and 6.1%, respectively. Distributions of hospitalization status, chronic comorbidities, and calendar month of specimen submitted were statistically different between the control and case groups.

**Table 1.** Baseline Characteristics of Cases and Controls Analyzed in the Interim Estimates of Vaccine Effectiveness Against Influenza From September 4, 2023, to March 25, 2024, 2023–2024 Influenza Season, Yinzhou in Southern China (n = 205 028)^a^

Characteristics	Test-Negative Controls	Any Influenza Cases		Influenza A Cases		Influenza B Cases	
	No. (%)	No. (%)	Cohen's *h*^b^	No. (%)	Cohen's *h*^b^	No. (%)	Cohen's *h*^b^
Total population	108 730 (100.0)	96 298 (100.0)	NA	64 988 (100.0)	NA	33 873 (100.0)	NA
Age groups	-	-	-	-	-	-	-
6 mo–6 y	16 644 (15.3)	13 763 (14.3)	0.03	10 040 (15.5)	0.001	4277 (12.7)	0.08
6–17 y	29 566 (27.2)	31 436 (32.6)	0.12	22 035 (33.9)	0.15	10 695 (31.7)	0.10
18–64 y	52 708 (48.5)	48 448 (50.3)	0.04	30 830 (47.4)	0.02	18 147 (53.9)	0.11
≥65 y	9812 (9.0)	2651 (2.8)	0.28	2083 (3.2)	0.25	579 (1.7)	0.35
Gender	-	-	-	-	-	-	-
Female	59 258 (54.5)	48 721 (50.6)	0.08	32 936 (50.7)	0.07	16 874 (50.1)	0.09
Male	49 472 (45.5)	47 577 (49.4)		32 052 (49.3)		16 824 (49.9)	
Vaccinated	-	-	-	-	-	-	-
Yes	14 628 (13.4)	7343 (7.6)	0.19	5476 (8.4)	0.16	2057 (6.1)	0.25
No	94 102 (86.6)	88 955 (92.4)		59 512 (91.6)		31 641 (93.9)	
Hospitalization status	-	-	-	-	-	-	-
Inpatient	3881 (3.6)	1419 (1.5)	0.14	1018 (1.6)	0.13	423 (9.8)	0.26
Outpatient or emergency	104 850 (96.4)	94 879 (98.5)		63 970 (98.4)		3880 (90.2)	
Chronic comorbidities^c^	-	-	-	-	-	-	-
No	89 449 (82.3)	83 178 (86.4)	0.11	54 606 (84.0)	0.05	30 709 (91.2)	0.26
Yes	19 281 (17.7)	13 120 (13.6)		10 382 (16.0)		2989 (8.9)	
Specimen submission month	-	-	-	-	-	-	-
September 2023	2850 (2.6)	426 (0.4)	0.24	413 (0.6)	0.18	38 (0.1)	0.27
October 2023	5309 (4.9)	2000 (2.1)	0.16	1950 (3.0)	0.10	152 (0.5)	0.30
November 2023	15 078 (13.9)	18 057 (18.8)	0.13	17 762 (27.3)	0.34	1032 (3.1)	0.41
December 2023	25 299 (23.3)	36 872 (38.3)	0.33	33 227 (51.1)	0.59	4752 (14.1)	0.24
January 2024	29 532 (27.21)	26 804 (27.8)	0.02	9333 (14.4)	0.31	17 840 (52.9)	0.53
February 2024	17 693 (16.3)	8829 (9.2)	0.21	1096 (1.7)	0.57	7763 (23.0)	0.17
March 2024^d^	12 969 (12.0)	3310 (3.4)	0.34	1207 (1.9)	0.43	2121 (6.3)	0.21

^a^The differences in all baseline characteristics between cases and controls were statistically significant, tested using the chi-square test.

^b^Cohen's *h* indicates the size of differences: negligible (<0.20), small (0.20–0.49), medium (0.50–0.79), and large (≥0.80).

^c^The chronic conditions included primary hypertension, all types of cancer, cardiovascular disease, chronic heart failure, cerebrovascular disease, chronic obstructive pulmonary disease, chronic kidney disease, diabetes, and neurodegenerative disorders.

^d^The data for specimens collected from March 26 to 31, 2024, were not included in this group.

### Overall Estimates of IVE

For the overall study population, adjusted IVE estimates in the 2023–2024 season against overall influenza, influenza A, and influenza B were 49.4% (95% CI, 43.8%–47.6%), 41.9% (95% CI, 41.9%–39.8%), and 59.9% (95% CI, 57.9%–61.9%), respectively (Table 2).

**Table 2.** Vaccine Effectiveness Against Influenza in the Total Study Population and Subgroups From September 4, 2023, to March 25, 2024, Yinzhou in Southern China^a^

	Test-Negative		Test-Positive		UnadjustedIVE (95% CI), %	AdjustedIVE (95% CI), %
Population	Unvaccinated,	Vaccinated,	Unvaccinated,	Vaccinated,		
	No. (%)	No. (%)	No. (%)	No. (%)		
Any influenza types	94 102 (86.6)	14 628 (13.5)	88 955 (92.4)	7343 (7.6)	46.9 (45.3, 48.4)	49.4 (47.8, 50.9)
Age groups	-	-	-	-	-	-
6 mo–6 y	12 204 (73.3)	4440 (26.7)	11 315 (82.2)	2448 (17.8)	40.5 (37.1–43.7)	45.8 (42.6–48.9)
7–17 y	24 273 (82.1)	5293 (17.9)	27 550 (87.6)	3886 (12.4)	35.3 (32.3–38.2)	38.6 (35.7–41.5)
18–64 y	51 860 (98.4)	848 (1.6)	48 149 (99.4)	299 (0.6)	62.0 (56.6–66.7)	46.7 (36.8–55.0)
≥65 y	5765 (58.7)	4047 (41.3)	1941 (73.2)	710 (26.8)	47.9 (42.7–52.6)	46.1 (33.7–48.7)
Chronic comorbidities	16 484 (85.5)	2797 (14.5)	12 074 (92.0)	1046 (8.0)	48.9 (45.0–52.6)	44.4 (39.9–48.5)
Hospitalization status	-	-	-	-	-	-
Inpatient	3035 (78.2)	845 (21.8)	1254 (88.4)	165 (11.6)	52.7 (43.5–60.5)	46.5 (35.4–55.8)
Outpatient and emergency	91 067 (86.9)	13 783 (13.2)	87 701 (92.4)	7178 (7.6)	45.9 (44.3, 47.5)	49.7 (48.1, 51.2)
Influenza A						
Overall	94 102 (86.6)	14 628 (13.5)	59 512 (91.6)	5476 (8.4)	40.8 (38.8–42.7)	41.9 (39.8–44.0)
Age groups	-	-	-	-	-	-
6 mo–6 y	12 204 (73.3)	4440 (26.7)	8111 (80.8)	1929 (19.2)	34.6 (30.6–38.5)	38.7 (34.6–42.6)
7–17 y	24 273 (82.1)	5293 (17.9)	19 294 (87.6)	2741 (12.4)	38.7 (31.5–38.0)	33.2 (29.4–36.8)
18–64 y	51 860 (98.4)	848 (1.6)	30 609 (99.3)	221 (0.7)	55.8 (48.8–61.9)	42.9 (32.6–51.5)
≥65 y	5765 (58.7)	4047 (41.3)	1498 (71.9)	585 (28.1)	44.4 (38.3–49.8)	40.4 (33.5–46.7)
Chronic comorbidities	16 484 (85.5)	2797 (14.5)	9501 (91.5)	881 (8.5)	45.4 (40.8–49.5)	39.9 (34.6–44.7)
Hospitalization status	-	-	-	-	-	-
Inpatient	3035 (78.2)	845 (21.8)	883 (86.7)	135 (13.3)	45.1 (33.2–54.9)	39.8 (25.8–51.1)
Outpatient and emergency	91 067 (86.9)	13 783 (13.2)	58 629 (91.7)	5341 (8.4)	39.8 (37.8–41.8)	42.1 (40.0–44.2)
Influenza B	94 102 (86.6)	14 628 (13.5)	31 641 (93.9)	2057 (6.1)	58.2 (56.1–60.9)	59.9 (57.9–61.9)
Age groups	-	-	-	-	-	-
6 mo–6 y	12 204 (73.3)	4440 (26.7)	3694 (86.4)	583 (13.6)	56.6 (52.4–60.5)	61.0 (57.0–64.6)
7–17 y	24 273 (82.1)	5293 (17.9)	9428 (88.2)	1267 (11.9)	48.4 (34.1–42.3)	48.4 (44.7–51.9)
18–64 y	51 860 (98.4)	848 (1.6)	18 066 (99.5)	81 (0.5)	72.5 (65.5–78.2)	67.3 (58.7–74.1)
≥65 y	5765 (58.7)	4047 (41.3)	453 (78.2)	126 (21.8)	60.4 (51.5–67.5)	62.6 (54.1–69.5)
Chronic comorbidities	16 484 (85.5)	2797 (14.5)	2811 (94.0)	178 (6.0)	62.7 (56.3–68.1)	62.9 (56.2–68.6)
Hospitalization status	-	-	-	-	-	-
Inpatient	3035 (78.2)	845 (21.8)	389 (92.0.)	34 (8.0)	68.6 (55.1–78.1)	60.5 (42.1–73.1)
Outpatient and emergency	91 067 (86.9)	13 783 (13.2)	31 252 (93.9)	2023 (6.1)	57.2 (55.1–59.3)	59.7 (57.6–61.7)

Odds ratios were derived from a univariate logistic model and a multivariate logistic regression model adjusted for age, gender, hospitalization status, presence of chronic conditions (yes or no), and the month when specimens were collected.

^a^Estimates of vaccine effectiveness was calculated as (1 – adjusted odds ratio) × 100%.

IVE estimates in high-risk subgroups and different clinical settings were also presented in Table 2. Vaccinated preschool children and elderly individuals, respectively, had a 45.8% (95% CI, 42.6%–48.9%) and 46.1% (95% CI, 33.7%–48.7%) lower risk of developing seasonal influenza. Among participants with chronic comorbidities, IVE was estimated to be 48.9% (95% CI, 45.0%–52.6%) against overall influenza, 45.4% (95% CI, 40.8%–49.5%) against influenza A, and 62.7% (95% CI, 56.3%–68.1%) against influenza B. IVE estimates were similar between inpatient and non-inpatient settings.

### Weekly Estimates of IVE

**Figure 2.**
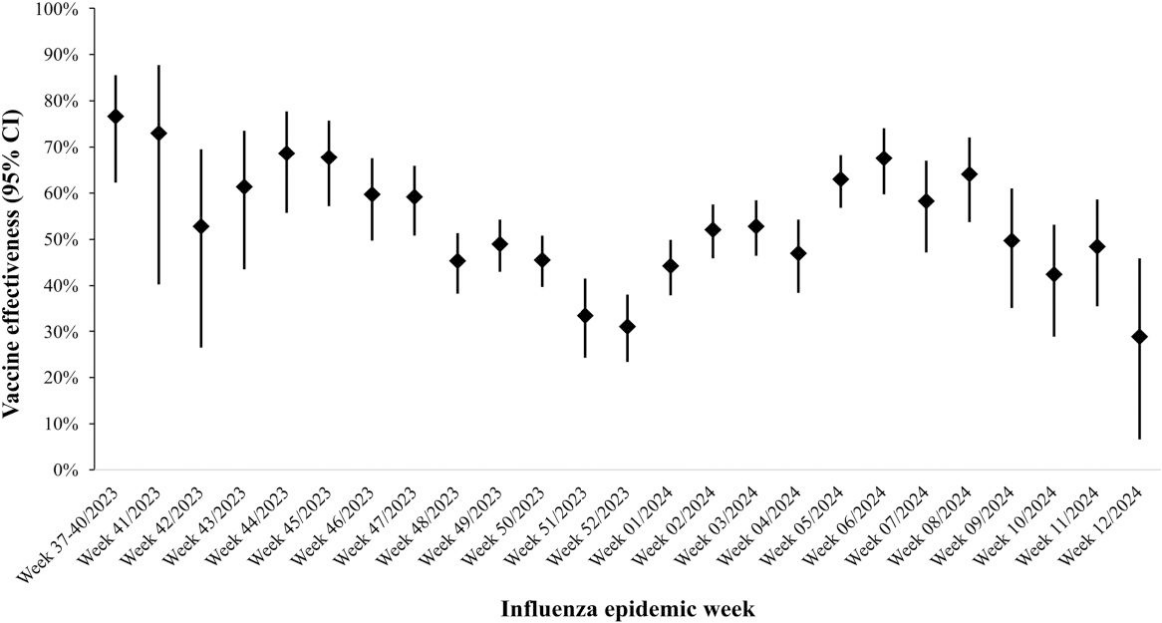
Weekly estimates of vaccine effectiveness against influenza infection from week 37/2023 to week 12/2024, 2023–2024 influenza season, Yinzhou, Zhejiang province, southern China.

The weekly estimates of IVE are shown in Figure 2. The estimate was high at the beginning of influenza season 2023–2024 (69.7%; 95% CI, 35.0%–85.9%) and decreased by the week 51/2024 (44.7%; 95% CI, 40.0%–49.0%). The estimates then started to increase in the first week of 2024.

### Sensitivity Analyses

Estimates of midseason vaccination effectiveness were 55.0% (95% CI, 51.9%–57.8%), 44.6% (95% CI, 40.1%–48.8%), and 67.4% (95% CI, 63.9%–70.6%) against all influenza types, influenza A, and influenza B, respectively (Supplementary Table 2). During the weeks when influenza A positivity rates were >10%, the IVE estimate was 42.7% (95% CI, 40.5%–44.8%). As for the higher influenza B activity period (positivity rate >10%), the IVE estimate was 60.7% (95% CI, 58.4%–62.8%) (Supplementary Table 3). The IVE estimates against overall influenza were 41.5% (95% CI, 39.5%–43.8%) among patients receiving the NAAT test and 39.4% (95% CI, 36.9%–41.8%) among patients receiving the RAT test (Supplementary Tables 4 and 5).

## DISCUSSION

Epidemic characteristics of 2023–2024 seasonal influenza in southern China and estimates of IVE against laboratory-confirmed influenza infection have been reported in this study. Using a test-negative case–control design, our study has shown that current influenza vaccines were effective in protecting against the circulating strains, with an overall IVE of 49.4%. However, estimates of IVE against influenza B (59.9%) were higher than those against influenza A (41.9%). Influenza vaccines also provided protection for high-risk subgroups, including preschool children (45.8%), elderly adults (46.1%), and participants with common chronic diseases (44.4%). We found that the weekly IVE peaked at around 70% protection against influenza infection in early September 2023 and early February 2024 when influenza A activities were relatively lower.

In the southern provinces of China, influenza virus detections peaked in December with a predominance of influenza A(H3N2) virus, followed by mostly B/Victoria virus since mid-January. The overall influenza activity trend in the CHERRY cohort was similar to that in sentinel hospitals of the southern provinces. These findings were different from observations in another East Asian country, South Korea, in which influenza A(H1N1)pdm09 was the predominant subtype [27].

To our knowledge, this study is the first population-based investigation on 2023–2024 seasonal IVEs against influenza infection in China. The IVE estimates in southern China are comparable to those in other Northern Hemisphere areas, including Europe and North America [28–30]. Our findings are also consistent with previous studies showing heterogeneity in IVE by influenza type [28–30]. Mismatching between vaccine compositions and circulating viruses is the major factor influencing IVE. It has been demonstrated that A(H3N2) virus evolves more frequently than B/Victoria [31]. Thus, the IVE is lower against A(H3N2) due to a higher chance of mismatch between vaccines and circulating A(H3N2). This could also explain the dynamic pattern of IVE throughout the season. We observed that weekly IVE estimates were lower during the A(H3N2)-dominated weeks and reached a bottom when levels of A(H3N2) activity peaked in December. IVE rates increased during the influenza B circulation weeks. However, in late March, IVE rates dropped dramatically, potentially because A(H1N1)pdm09 became the dominant subtype. According to data from South Korea, estimates of IVE against A(H1N1)pdm09 were relatively low (22.5%) [27]. Moreover, previous studies have shown a waning IVE since the time when influenza vaccine administrated during the season [32, 33]. A Canadian study reported that the odds of influenza increased by 9% every 28 days after vaccination [32]. Another study conducted in a tropical climate (which has year-round influenza activity) demonstrated that the odds of infection increased 7% every 56 days after vaccination [33]. This could partially explain the decreasing weekly IVE observed in our study. Further studies are recommended to investigate the waning protection of the influenza vaccine with long-term observational data in the Chinese population.

It is noteworthy that we observed lower IVE rates in individuals aged 7–17 years compared with other age groups, especially for IVE against B/Victoria (48.4% vs 61.0%–67.3%). Similarly, Hood et al. have shown that IVE was significantly lower for children aged 5 to 17 years compared with children aged 6 to 59 months [34]. However, it is often reported that IVE for children and teenagers is higher compared with IVE for adults [10, 28–30]. Heavier exposure to influenza could be a factor leading to the lower estimate of IVE [35]. In our study, vaccine coverage was relatively lower (17.9% in controls and 12.4% in cases) among individuals aged 7–17 years, who usually cluster in schools. The burden of influenza activity in this age group was heavier, which could be the reason for lower IVE estimates. Therefore, promoting influenza vaccines is recommended to improve vaccination coverage among school children during influenza seasons.

Influenza vaccines provided protection against influenza among adults aged ≥65 years in the 2023–2024 season. However, the point estimates of IVE for adults aged ≥65 years were slightly lower compared with the point estimates of IVE for younger adults. This finding is in line with the evidence from previous studies [10, 28–30]. Aging is widely recognized to have a negative impact on both the innate and adaptive immune systems, leading to a decline in immune response known as immunosenescence, which can reduce IVE [36]. Various strategies could be implemented to enhance protection against influenza among the elderly, such as improving immunization coverage, increasing the dose of hemagglutinin antigen, and adding adjuvants to vaccines [36].

In southern China, the mass vaccination campaign against influenza usually runs from early August to late February of the following year. Vaccination before the start of influenza season (influenza test positivity rate >10%) is encouraged [37]. Nevertheless, for individuals who were not vaccinated in the months before the influenza season, our data suggest that vaccination within the season still conferred protection for the entire population.

A key strength of our study was the timely estimation of IVE. Based on the Yinzhou Healthcare Big Data Platform, the CHERRY cohort integrates comprehensive data in the health care system on a real-time basis, which makes it possible to conduct a near-real-time estimation of seasonal IVE in the entire population of the district [38, 39]. In-season IVE is of significant importance during severe influenza seasons, especially in the presence of virological mismatches or pandemics. Based on this platform, a weekly surveillance framework for seasonal IVE will be established to inform public health authorities in policy planning and adjustment, as well as resource allocation. Moreover, previous Chinese population–based IVE studies were unable to evaluate IVE in individuals aged 9–60 years and by influenza type [40]. The large sample size of our study allowed for stratified analyses of IVE according to influenza type, age group, and disease-specific population. Additionally, combined with epidemiological history and clinical manifestations, both NAATs and RATs can be used for influenza diagnosis in China's clinical practice. Therefore, in the CHERRY cohort, a great number of influenza cases were confirmed by RATs. Our sensitivity analyses indicated that the IVE derived from NAAT data was comparable with the IVE derived from RAT data (41.5% vs 39.4%).

Several limitations need to be considered when interpreting the findings. First, specific types and compositions of vaccines were not available for individuals in the CHERRY cohort. There are 3 vaccines, trivalent inactivated vaccine, trivalent live attenuated vaccine, and quadrivalent inactivated vaccine, available in China [41]. According to the WHO's recommendations, vaccine compositions were applied for the 2023‒2024 season. The compositions are the same between the trivalent vaccine and quadrivalent vaccine, except for B/Yamagata in the quadrivalent vaccine. B/Yamagata was rarely detected in this influenza season. Therefore, IVEs of trivalent vaccine and quadrivalent vaccine should be similar. Second, the influenza vaccine dosage for each individual was not available. Without this information, it is challenging to determine variations in vaccine effectiveness related to dosage, particularly for the elderly who are recommended to receive high-dose influenza vaccines. Third, the influenza A subtypes and B lineages were detected in a small group of patients, and the sample size is not sufficient for IVE estimation. Therefore, IVE against different subtypes and lineages was not included in this study.

## CONCLUSIONS

Given the fact that multiple pathogens resurged after the relaxation of COVID-19 restrictions, there were considerable uncertainties surrounding IVE in communities. Our findings based on a large population-based cohort indicated that vaccination has been protective against 2023–2024 seasonal influenza in the Chinese context. Nonetheless, the public health benefit of influenza vaccination programs is determined by both IVE and the vaccination coverage rate. Increasing vaccination coverage will improve the prevention of influenza infections and related illnesses, especially for vulnerable populations.

## Supplementary Material

ofae456_Supplementary_Data

## Supplementary Data

Supplementary materials are available at *Open Forum Infectious Diseases* online. Consisting of data provided by the authors to benefit the reader, the posted materials are not copyedited and are the sole responsibility of the authors, so questions or comments should be addressed to the corresponding author.
