# Supplementary material for: Population-Based Influenza Vaccine Effectiveness Against Laboratory-Confirmed Influenza Infection in Southern China, 2023–2024 Season
Source: Open Forum Infect Dis. 2024 Aug 21;11(9):ofae456. doi: 10.1093/ofid/ofae456 (PMC11365065; doi:10.1093/ofid/ofae456)
Supplement: ofae456_Supplementary_Data [file ofae456_supplementary_data.docx]

**Supplementary Materials**

**List of contents**

**Table S1** ICD-10 codes utilized to define major chronic comorbidities

**Table S2** Adjusted mid-season vaccination effectiveness against laboratory-confirmed influenza in season 2023/24a b

**Table S3** Adjusted IVE against laboratory–confirmed influenza during higher influenza A activity period and during higher influenza B activity perioda

**Table S4** Adjusted influenza vaccine effectiveness estimates against NAAT–confirmed influenza in season 2023–24

**Table S5** Adjusted influenza vaccine effectiveness estimates against RAT–confirmed influenza in season 2023–24a

**Figure S1** Weekly number of influenza-positive specimens and positivity rate from week 37/2023-week 12/2024, 2023-24 influenza season, Yinzhou, southern China

| **Table S1** ICD-10 codes utilized to define major chronic comorbidities | |
| --- | --- |
| **Comorbidity** | **ICD-10 codes** |
| Neoplasm | C00-C96, D00-D48 |
| Diabetes mellitus | E10-E14 |
| Hypertension | I10 |
| Cardiovascular diseases | I20-I25 |
| Congestive heart failure | I50 |
| Cerebrovascular diseases | I60-I69 |
| Chronic kidney disease | N18 |
| Chronic obstructive pulmonary disease | J44 |
| Neurodegerative diseases | F00-F03, G12, G20, G30 |

Abbreviation: ICD-10: International Classification of Diseases 10th Revision

| **Table S2** Adjusted mid-season vaccination effectiveness against laboratory-confirmed influenza in season 2023/24**^a b^** | | | |
| --- | --- | --- | --- |
|  | **Any influenza** | **Influenza A** | **Influenza B** |
|  | **VE (95% CI), %** | **VE (95% CI), %** | **VE (95% CI), %** |
| **Overall** | 55.0 (51.9–57.8) | 44.6 (40.1–48.8) | 67.4 (63.9–70.6) |
| **Age groups** | - | - | - |
| 6 months–6 years | 41.8 (46.6–56.5) | 40.4 (33.0–47.1) | 68.5 (62.5–73.5) |
| 7–17 years | 49.7 (44.7–54.3) | 41.3 (33.8–47.9) | 60.0 (54.0–65.2) |
| 18–64 years | 47.5 (35.8–57.1) | 41.3 (33.8–47.9) | 63.5 (49.4–73.7) |
| ≥ 65 years | 43.6 (–4.5–69.5) | 31.7 (-35.1–65.4) | 72.9 (-11.9–93.4) |
| **With major chronic comorbidities** | 51.8 (41.1–60.5) | 44.1 (30.2–55.2) | 74.0 (62.0–82.1) |
| **Hospitalization status** | - | - | - |
| Inpatient | 52.3 (9.9–74.8) | 37.3 (-30.3–69.9) | 69.8 (10.1–89.8) |
| Outpatient and emergency | 54.9 (51.8, 57.8) | 44.7 (40.2–48.8) | 67.3 (63.7–70.5) |

Abbreviation: CI, confidence interval; VE: vaccine effectiveness.

**a** Mid-season vaccination was defined as the vaccination date later than the start of the influenza season.

**b** Estimates of vaccine effectiveness was calculated as (1 – adjusted odds ratio) × 100%. Odds ratios were derived from multivariate logistic regression model adjusted for age, gender, hospitalization status, presence of chronic conditions (yea or no), and the month when specimens were collected.

| **Table S3** Adjusted IVE against laboratory–confirmed influenza during higher influenza A activity period and during higher influenza B activity period^a^ | | |
| --- | --- | --- |
|  | **Influenza A** | **Influenza B** |
|  | **VE (95% CI), %** | **VE (95% CI), %** |
| **Overall** | 42.7 (40.5–44.8) | 60.7 (58.4–62.8) |
| **Age groups** | - | - |
| 6 months–6 years | 40.3 (36.0–44.4) | 59.4 (54.8–63.6) |
| 7–17 years | 33.7 (29.8–37.4) | 47.4 (43.1–51.3) |
| 18–64 years | 43.5 (32.4–52.8) | 67.3 (58.3–74.3) |
| ≥ 65 years | 41.2 (33.2–48.2) | 61.7 (52.7–68.9) |
| **With major chronic comorbidities** | 39.7 (34.2–44.7) | 61.0 (53.2–67.4) |
| **Hospitalization status** | - | - |
| Inpatient | 42.4 (27.1–54.5) | 58.5 (38.6–72.0) |
| Outpatient and emergency | 42.8 (40.5–45.0) | 60.4 (58.2–62.6) |

Abbreviation: CI, confidence interval; VE: vaccine effectiveness.

**a** Estimates of vaccine effectiveness was calculated as (1 – adjusted odds ratio) × 100%. Odds ratios were derived from multivariate logistic regression model adjusted for age, gender, hospitalization status, presence of chronic conditions (yea or no), and the month when specimens were collected.

| **Table S4** Adjusted influenza vaccine effectiveness estimates against NAAT–confirmed influenza in season 2023–24 | | | |
| --- | --- | --- | --- |
|  | **Any influenza** | **Influenza A** | **Influenza B** |
|  | **VE (95% CI), %** | **VE (95% CI), %** | **VE (95% CI), %** |
| **Overall** | 41.5 (39.5–43.8) | 48.7 (45.9–51.6) | 68.2 (65.3–70.8) |
| **Age groups** | - | - | - |
| 6 months–6 years | 41.8 (37.4–46.6) | 49.2 (43.6–55.5) | 74.3 (68.8–78.9) |
| 7–17 years | 52.4 (48.3–56.7) | 55.8 (50.9–61.3) | 54.6 (48.8–59.7) |
| 18–64 years | 37.1 (30.6–44.9) | 52.1 (40.0–61.7) | 77.0 (67.6–83.7) |
| ≥ 65 years | 59.2 (52.0–67.4) | 34.8 (24.8–43.5) | 61.3 (48.4–71.0) |
| **With major chronic comorbidities** | 50.6 (45.1–56.9) | 47.0 (40.0–53.2) | 65.7 (55.8–73.4) |
| **Hospitalization status** | - | - | - |
| Inpatient | 54.8 (44.7–67.3) | 38.5 (23–58.1) | 61.1 (40.5–74.6) |
| Outpatient and emergency | 40.9 (38.8, 43.1) | 52.5 (49.5–55.3) | 68.0 (65.0–70.7) |

Abbreviation: CI, confidence interval; VE: vaccine effectiveness.

**a** Estimates of vaccine effectiveness was calculated as (1 – adjusted odds ratio) × 100%. Odds ratios were derived from multivariate logistic regression model adjusted for age, gender, hospitalization status, presence of chronic conditions (yea or no), and the month when specimens were collected.

| **Table S5** Adjusted influenza vaccine effectiveness estimates against RAT–confirmed influenza in season 2023–24**^a^** | | | |
| --- | --- | --- | --- |
|  | **Any influenza** | **Influenza A** | **Influenza B** |
|  | **VE (95% CI), %** | **VE (95% CI), %** | **VE (95% CI), %** |
| **Overall** | 39.4 (36.9–41.8) | 30.7 (27.3–33.9) | 51.7 (48.6–54.7) |
| **Age groups** | - | - | - |
| 6 months–6 years | 40.8 (36.6–44.8) | 34.4 (29.1–39.4) | 54.7 (49.2–59.6) |
| 7–17 years | 36.6 (32.8–40.3) | 30.8 (25.6–35.5) | 46.8 (41.9–51.2) |
| 18–64 years | 41.1 (27.8–52.0) | 31.6 (12.3–46.7) | 54.4 (37.1–67.0) |
| ≥ 65 years | 37.4 (26.4–46.7) | 26.6 (11.6–39.2) | 56.9 (41.9–68.1) |
| **With major chronic comorbidities** | 31.3 (23.4–38.4) | 22.1 (12.2–30.8) | 58.6 (48.1–66.9) |
| **Hospitalization status** | - | - | - |
| Inpatient | 41.9 (2.1–65.6) | 34.0 (-19.3–69.4) | 46.5 (-31.8–78.3) |
| Outpatient and emergency | 39.4 (36.9–41.7) | 30.7 (27.3–33.9) | 51.7 (48.5–54.6) |

Abbreviation: CI, confidence interval; VE: vaccine effectiveness.

**a** Estimates of vaccine effectiveness was calculated as (1 – adjusted odds ratio) × 100%. Odds ratios were derived from multivariate logistic regression model adjusted for age, gender, hospitalization status, presence of chronic conditions (yea or no), and the month when specimens were collected.

**
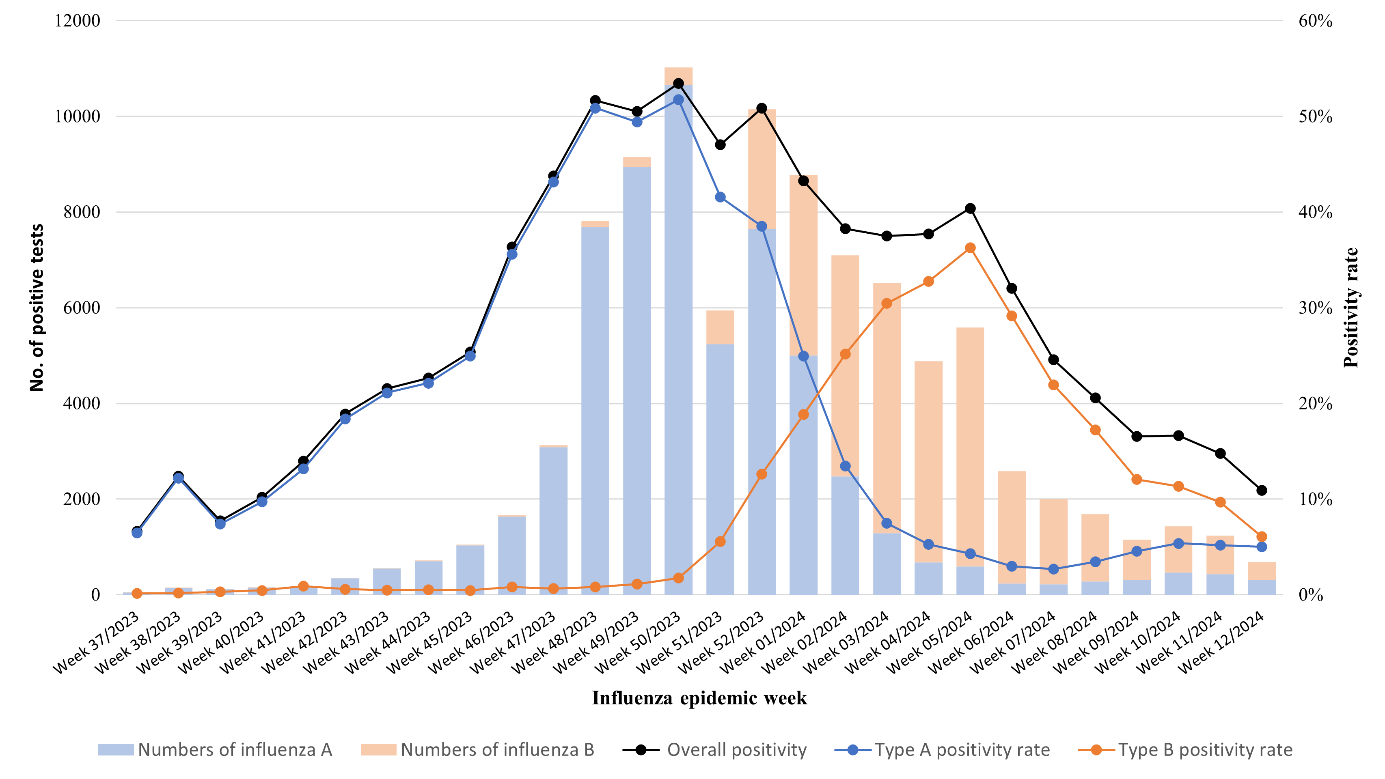
**

**Figure S1** Weekly number of influenza-positive specimens and positivity rate from week 37/2023-week 12/2024, 2023-24 influenza season, Yinzhou, southern China
